# Supplementary material for: Ordinary Gasoline Emissions Induce a Toxic Response in Bronchial Cells Grown at Air-Liquid Interface
Source: Int J Mol Sci. 2020 Dec 23;22(1):79. doi: 10.3390/ijms22010079 (PMC7801947; doi:10.3390/ijms22010079)
Supplement: Supplementary file 1 [file ijms-22-00079-s001.zip › Supplementary File 2.docx]

Verification of mRNA sequencing expression data using qRT-PCR: a list of selected genes and results of qRT-PCR analysis

| Gene name | Ensembl ID | Log2FC | p-value |
| --- | --- | --- | --- |
| CYP1A1 | ENSG00000140465 | -2.039 | <0.05 |
| BID | ENSG00000015475 | -0.021 | 0.851 |
| PCNA | ENSG00000132646 | -0.137 | 0.165 |
| IL6 | ENSG00000136244 | -1.120 | <0.001 |
| PTGS2 | ENSG00000073756 | -1.400 | <0.001 |

TaqMan assays (Thermo Fisher Scientific, Waltham, MA USA) used for qRT-PCR verification of mRNA expression data by mRNA sequencing

| **ID** | **Gene name** |
| --- | --- |
| Hs00153120_m1 | CYP1A1 |
| Hs00609632_m1 | BID |
| Hs00696862_m1 | PCNA |
| Hs00985639_m1 | IL6 |
| Hs00153133_m1 | PTGS2 |
| Hs00243257_m1 | TOP1 |
| Hs01060665_g1 | ACTB |
